# EIF5A1 promotes trophoblast migration and invasion via ARAF-mediated activation of the integrin/ERK signaling pathway

**DOI:** 10.1038/s41419-018-0971-5

**Published:** 2018-09-11

**Authors:** Jing Zhang, Hui-Qin Mo, Fu-Ju Tian, Wei-Hong Zeng, Xiao-Rui Liu, Xiao-Ling Ma, Xiao Li, Shi Qin, Cui-Fang Fan, Yi Lin

**Affiliations:** 1 International Peace Maternity & Child Health Hospital, School of Medicine, Shanghai Jiao Tong University, Shanghai, P. R. China; 2 Institute of Embryo-Fetal Original Adult Disease Affiliated to Shanghai Jiao Tong University School of Medicine, Shanghai, P. R. China; 3 Department of Obstetrics and Gynecology, Renmin Hospital of Wuhan University, Wuhan, P. R. China

## Abstract

Trophoblast dysfunction is one mechanism implicated in the etiology of recurrent miscarriage (RM). Regulation of trophoblast function, however, is complex and the mechanisms contributing to dysregulation remain to be elucidated. Herein, we found EIF5A1 expression levels to be significantly decreased in cytotrophoblasts in RM villous tissues compared with healthy controls. Using the HTR-8/SVneo cell line as a model system, we found that overexpression of EIF5A1 promotes trophoblast proliferation, migration and invasion in vitro. Knockdown of EIF5A1 or inhibiting its hypusination with N1-guanyl-1,7-diaminoheptane (GC7) suppresses these activities. Similarly, mutating EIF5A1 to EIF5A1_K50A_ to prevent hypusination abolishes its effects on proliferation, migration and invasion. Furthermore, upregulation of EIF5A1 increases the outgrowth of trophoblasts in a villous explant culture model, whereas knockdown has the opposite effect. Suppression of EIF5A1 hypusination also inhibits the outgrowth of trophoblasts in explants. Mechanistically, ARAF mediates the regulation of trophoblast migration and invasion by EIF5A1. Hypusinated EIF5A1 regulates the integrin/ERK signaling pathway via controlling the translation of ARAF. ARAF level is also downregulated in trophoblasts of RM villous tissues and expression of ARAF is positively correlated with EIF5A1. Together, our results suggest that EIF5A1 may be a regulator of trophoblast function at the maternal–fetal interface and low levels of EIF5A1 and ARAF may be associated with RM.

## Introduction

Recurrent miscarriage (RM), an important reproductive issue, is defined as two or more clinical pregnancy losses before 20 weeks of gestation according to the American Society for Reproductive Medicine^1^. Approximately 2–5% of childbearing couples suffer from RM and the associated psychological distress each year^2,3^. Historically, the etiology of RM has been attributed to either genetic causes, uterine structural abnormalities, infection, endocrine factors, immune factors, inherited thrombophilias or unexplained causes^4,5^. Although new interventions to treat RM clinically show some success, >25% of patients remain unable to have a successful subsequent pregnancy due to the lack of effective treatment strategies^6^. An understanding of the pathogenesis of RM, especially unexplained RM, and the development of targeted therapeutic approaches is essential to improve the rate of successful pregnancies in RM patients.

Trophoblasts are the most important cells of the placenta in embryo implantation and formation of the maternal–fetal interface^7^. There are three types of trophoblasts: cytotrophoblasts (CTBs), CTB differentiated to syncytiotrophoblast (STB), and extravillous trophoblasts (EVTs)^8^. Dysfunction of EVTs, which have a highly invasive character, may cause a series of pregnancy complications including pre-eclampsia, fetal growth restriction and RM^7^. The regulation of trophoblast proliferation, migration and invasion involves a variety of factors in the maternal–fetal interface including growth factors, growth factor-binding proteins and extracellular matrix (ECM), which trigger complex signaling pathways^9^. It is reported that the insulin-like growth factor-binding protein-1 stimulates trophoblast migration through integrin α5β1 via the mitogen-activated protein kinase (MAPK) pathway^10^. Integrins regulate trophoblast invasion through focal adhesion kinase (FAK)-dependent or -independent activation of the Extracellular Signal-regulated Kinase (ERK) signaling pathway^11,12^. Previous work from our group indicated that the YY1/MMP2 axis promotes trophoblast invasion at the maternal–fetal interface and downregulation of YY1 is associated with RM^13^. Consequently, studying the complex regulatory networks of trophoblasts may contribute to clarifying the pathogenesis of RM.

Eukaryotic translation initiation factor 5A1 (*EIF5A1*), located on chromosome 17p13.1, encodes a protein highly conserved during eukaryotic evolution. In a reaction catalyzed by deoxyhypusine synthase (DHS) and deoxyhypusine hydroxylase, the lysine at position 50 (K50) of EIF5A1 is subject to post-translational hypusination (polyamine-derived amino-acid hypusine, Nε-[4-amino-2-hydroxybutyl]-lysine)^14^. GC7 (N1-guanyl-1,7-diaminoheptane) is a specific inhibitor of DHS that has been used to suppress the hypusination of EIF5A1 to inhibit its functions in many studies^15^. To date, the functions of EIF5A1 identified in eukaryotic cells include RNA and ribosome binding, shuttling between the nucleus and cytoplasm, exporting mRNA from the nucleus to the cytoplasm, and translation elongation and termination^16–18^. EIF5A1 has been reported as an enhancer of cell proliferation, migration and invasion in some cancers including ovarian cancer, cervical carcinoma and pancreatic cancer^19–21^. Our previous studies have shown that EIF5A1 levels are lower in uterine lymphocytes derived from non-obese diabetic mice with impaired fertility than in wild-type (WT) mice, and inhibition of EIF5A1 with GC7 results in uterine natural killer cell dysfunction and embryo loss in mice^22,23^. However, it is unknown whether EIF5A1 participates in the regulation of trophoblast biology.

In this study, we found that EIF5A1 is downregulated in trophoblasts of RM villous tissues. EIF5A1 regulates trophoblasts proliferation, migration, invasion in vitro and outgrowth in an explant culture model. To regulate these processes, EIF5A1 requires the hypusine modification. Then hypusinated EIF5A1 activates the integrin/ERK signaling pathway via controlling the translation of ARAF mRNA. ARAF is also downregulated in trophoblasts of RM samples and expression is positively correlated with EIF5A1. Together, our study suggests that EIF5A1 and ARAF may be involved in the pathogenesis of RM.

## Results

### EIF5A1 is downregulated in CTBs from RM tissues

**Fig. 1:**
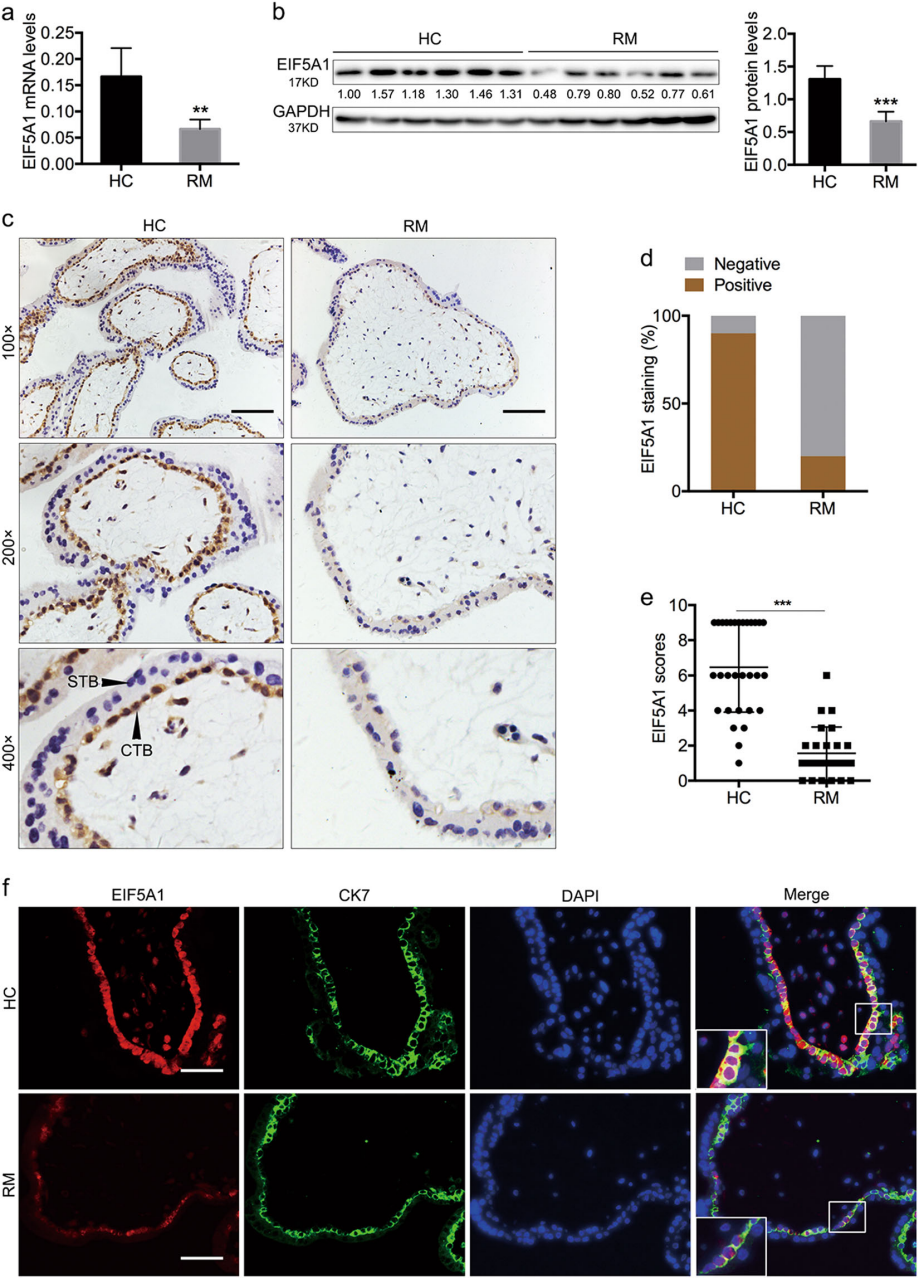
EIF5A1 is downregulated in cytotrophoblasts (CTBs) from recurrent miscarriage (RM) tissues. **a** EIF5A1 mRNA levels in villous tissues. **b** EIF5A1 protein levels in villous tissues. GAPDH served as a loading control. **c** Representative images of IHC staining of EIF5A1 in RM and healthy control (HC) samples (scale bar = 100 μm). CTBs and syncytiotrophoblasts (STBs) are indicated by arrows. **d** The percentage of EIF5A1-positive cases based on IHC staining. **e** IHC staining scores for EIF5A1. **f** Double IF staining of EIF5A1 (red) and CK7 (green) in villous tissues of RM and HC samples (magnification × 200; scale bar = 50 μm). **P < 0.01, ***P < 0.001

To evaluate EIF5A1 expression in first-trimester chorionic villous tissues, we performed real-time PCR and western blotting assays on 12 human samples (6 RM samples and 6 healthy controls [HCs]). The results show that both mRNA (Fig. 1a) and protein (Fig. 1b) levels of EIF5A1 are decreased in RM samples compared with HCs. Furthermore, immunohistochemical (IHC) staining of 25 RM and 30 HC first-trimester villous samples revealed that EIF5A1 expression is weaker in RM samples compared with HCs (Fig. 1c–e). IHC also indicated that EIF5A1 is mainly expressed in the CTB layer but not in STBs. To further verify the expression levels and localization of EIF5A1, we conducted a double immunofluorescence (IF) procedure to examine the trophoblast marker cytokeratin 7 (CK7) and EIF5A1 in 25 RM and 30 HC samples (Fig. 1f). The results show that EIF5A1 is only detected in CTBs and is more strongly expressed in HCs than RM samples. The low expression of EIF5A1 in CTBs from RM patients suggests that it could be involved in the pathogenesis of RM.

### EIF5A1 promotes trophoblasts proliferation, migration and invasion in vitro and outgrowth in a villous explant culture model

**Fig. 2:**
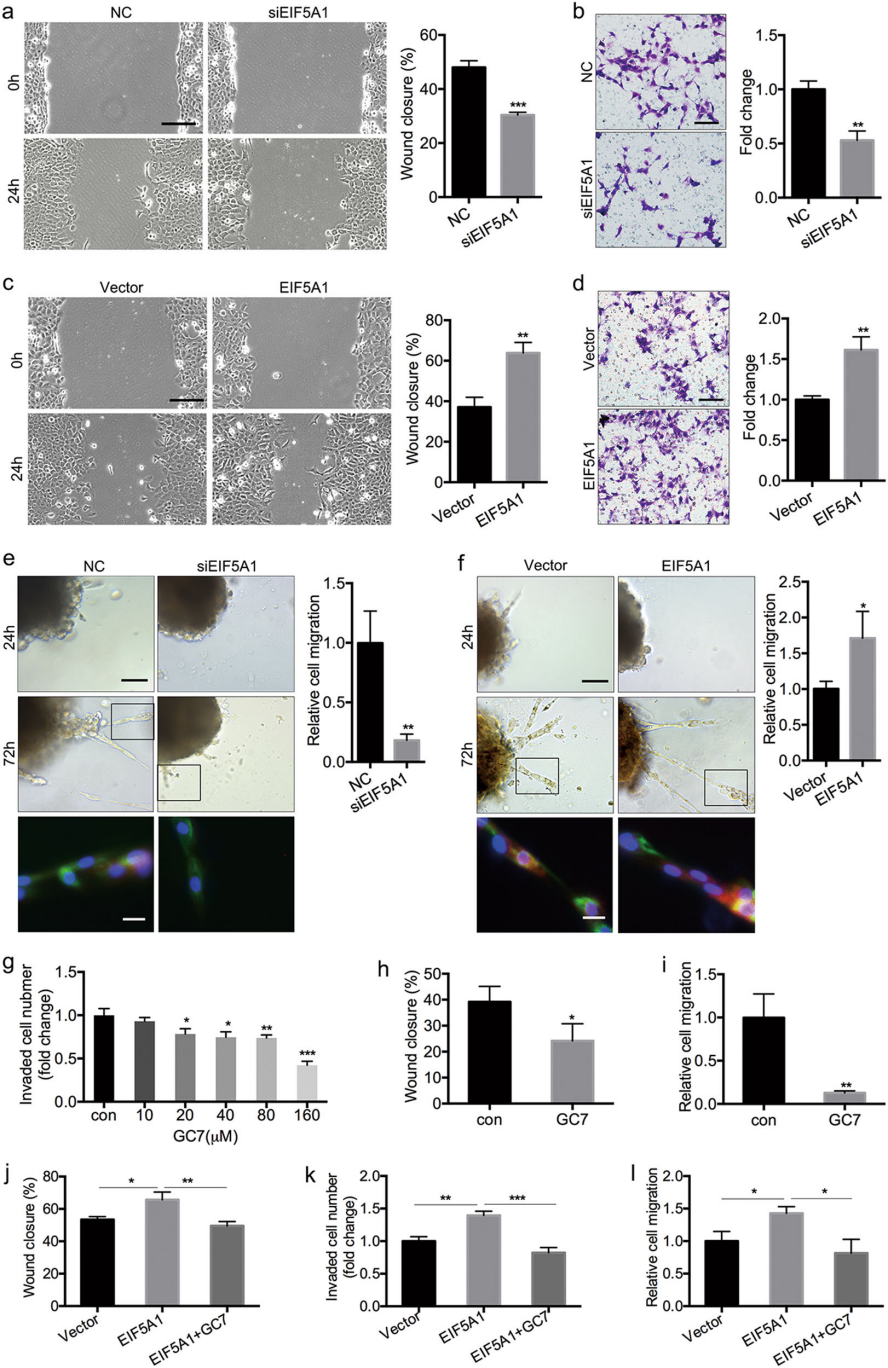
EIF5A1 promotes trophoblast migration and invasion in vitro and outgrowth in a villous explant culture model. **a** Wound-healing assay showing the effects of EIF5A1 knockdown on HTR-8 cells migration. Original magnification × 100, scale bar = 200 μm. **b** Invasion assay showing that downregulation of EIF5A1 affects HTR-8 invasion. Original magnification × 200, scale bar = 100 μm. **c** Wound-healing assays demonstrating the effects of EIF5A1 upregulation on HTR-8 migration. Original magnification × 100, scale bar = 200 μm. **d** Invasion assay showing that overexpression of EIF5A1 promotes HTR-8 invasion. Original magnification × 200, scale bar = 100 μm. **e-f** Villous explants were obtained from healthy controls at 8–10 weeks of gestation and cultured on Matrigel. siEIF5A1 (**e**) or EIF5A1 (**f**) were transfected into the tissues. Images were acquired after in vitro culture for 24 h and 72 h. Original magnification × 200, scale bar = 100 μm. Immunofluorescence (IF) images of trophoblasts were expressing EIF5A1 (red) and CK7 (green). Original magnification × 200, scale bar = 25 μm. **g** The fold change in the number of invaded HTR-8 cells after treatment with a gradient of GC7 for 24 h. **h** Would-healing assay showing control (con) and GC7-treated HTR-8 cells. **i** Relative migration of trophoblasts in villous explants after GC7 treatment. **j-k** Changes in migratory (**j**) and invasive (**k**) capabilities in EIF5A1-overexpressing HTR-8 cells after incubation with GC7. **l** Effects of GC7 on trophoblast outgrowth induced by EIF5A1 overexpression in villous explants. GC7 treatment was performed at a concentration of 160 μM for 24 h. *P < 0.05, **P < 0.01, ***P < 0.001

To investigate the role of EIF5A1 in modulating trophoblast biology, we transfected HTR-8/SVneo (HTR-8) cells with small interfering RNA (siRNA) or an EIF5A1 expression plasmid to achieve knockdown or overexpression, respectively (Figure S1a). The MTS [3-(4,5-Dimethylthiazol-2-yl)-5-(3-carboxymethoxyphenyl)-2-(4-sulfophenyl)-2H-tetrazolium] proliferation assay showed that knockdown or overexpression of EIF5A1 suppresses or promotes HTR-8 cell proliferation, respectively (Figure S1b). Next, wound-healing and Matrigel transwell assays were performed to evaluate the migratory and invasive capacities of trophoblasts. In EIF5A1 knockdown cells, wound closure is delayed and the number of invading cells is decreased compared with the negative control (Fig. 2a, b). In contrast, wound closure is accelerated and invading cell numbers are increased in EIF5A1-overexpressing cells compared with the negative control (Fig. 2c, d).

Villous explants derived from 8 to 10 weeks healthy gestation samples cultured on Matrigel-coated plates were used to verify the role of EIF5A1 in trophoblast migration ex vivo. Migratory ability was measured by trophoblast outgrowth on the Matrigel surface at 24 h and 72 h. The results show that downregulation or upregulation of EIF5A1 enhances or weakens the migratory ability of trophoblasts, respectively (Fig. 2e, f).

These results indicate that EIF5A1 promotes trophoblasts proliferation, migration and invasion in vitro and ex vivo.

### EIF5A1 regulation of trophoblast proliferation, migration and invasion is dependent on hypusination

GC7 is a specific inhibitor of EIF5A1 hypusination. EIF5A1 protein levels in HTR-8 cells show no obvious change following treatment with a gradient of GC7 concentrations (Figure S2a). GC7 suppresses HTR-8 proliferation in a dose-dependent manner at 12 h, 24 h or 48 h as determined by MTS assay (Fig. S2b). GC7 also inhibits HTR-8 invasion capacity in a dose-dependent manner (Fig. 2g and Figure S2c). Based on the inhibitory effects on proliferation and invasion, we chose a concentration of 160 µM and treatment time of 24 h for follow-up studies. Wound-healing assays performed in the presence of GC7 show that GC7 delays wound closure in HTR-8 cells (Fig. 2h and Figure S2d). Consistent with the in vitro migration assay, GC7 similarly reduces trophoblasts outgrowth in a villous explant culture model (Fig. 2i and Figure S2e).

As suppressing hypusination with GC7 inhibits proliferation, migration and invasion of trophoblasts, we speculated that EIF5A1 regulation of these processes relies on its hypusination. To explore this hypothesis, we performed rescue assays. As shown in Fig. 2j, k and Figure S3a, b, the promotion of HTR-8 cell migration and invasion achieved by EIF5A1 overexpression is attenuated by treatment with 160 µM GC7 for 24 h. GC7 treatment similarly restrained trophoblast outgrowth on a Matrigel surface induced by EIF5A1 upregulation (Fig. 2l and Figure S3c). We constructed an EIF5A1_K50A_ plasmid bearing a single point mutation (K50 → A50) that prevents hypusination. We then transfected HTR-8 cells with this plasmid. MTS, wound-healing and Matrigel transwell assays were used to measure proliferative, migratory and invasive abilities, respectively. We found each of these abilities to be decreased in EIF5A1_K50A_-transfected HTR-8 cells as compared with EIF5A1-transfected cells, with levels comparable to the vector control (Figure S4a–c). Therefore, we confirmed that EIF5A1 hypusination is indispensable in promoting trophoblast proliferation, migration and invasion.

### EIF5A1 regulates ARAF protein expression in trophoblasts via directly binding to its mRNA

**Fig. 3:**
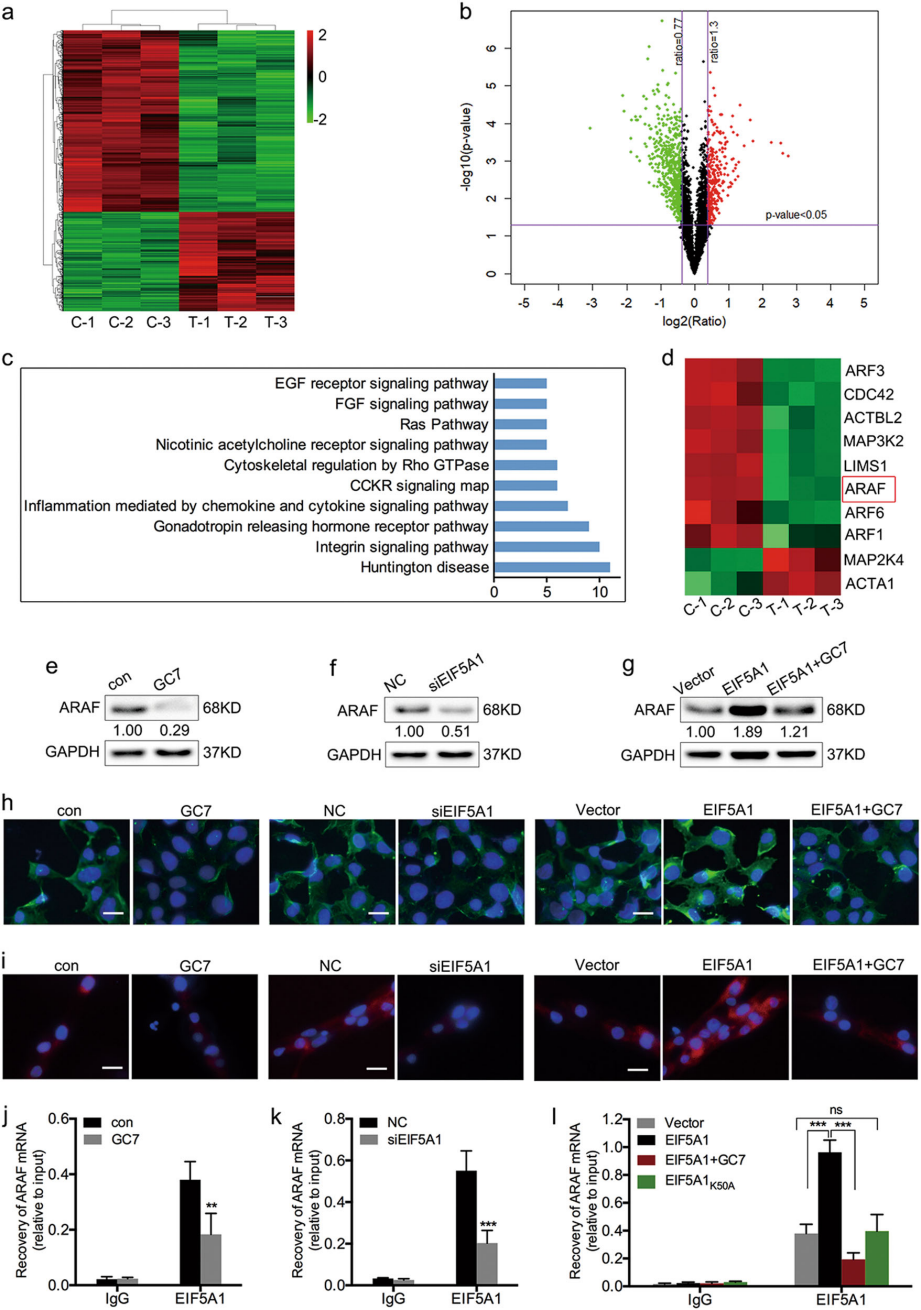
EIF5A1 regulates ARAF expression in trophoblasts through directly binding to its mRNA. **a, b** Heatmap (**a**) and volcano plot (**b**) showing differentially expressed proteins after GC7 treatment. C1–3 indicates control samples, T1–3 indicate treated samples. **c** Signaling pathways enriched following GC7 treatment based on PANTHER analyses. **d** Heatmap showing expression of the 10 dysregulated proteins from the integrin signaling pathway. **e, f** The effects of GC7 (**e**) and siEIF5A1 (**f**) treatment on ARAF protein expression in HTR-8 cells as evaluated by western blotting. **g** Western blot showing the effects of EIF5A1 upregulation or GC7 treatment on ARAF expression in naive HTR-8 or EIF5A1-overexpressing HTR-8 cells. **h** IF staining of ARAF in HTR-8 cells treated with GC7, siEIF5A1, EIF5A1, EIF5A1 + GC7 and their controls. Original magnification × 200, scale bar = 25 μm. **i** IF staining of ARAF in trophoblasts of villous explants treated with GC7, siEIF5A1, EIF5A1, EIF5A1 + GC7 and their controls. Original magnification × 200, scale bar = 25 μm. **j-l** RIP assays from HTR-8 cells treated with GC7, siEIF5A1, EIF5A1, EIF5A1 + GC7 and EIF5A1_K50A_. The cell lysates were incubated with either the EIF5A1 antibody or IgG. **P < 0.01, ***P < 0.001

To further explore the mechanism through which hypusinated EIF5A1 regulates trophoblast proliferation, migration and invasion, three paired HTR-8 cell samples treated with 160 µM GC7 or vehicle for 24 h were collected and subjected to the isobaric tags for relative and absolute quantitation (iTRAQ) labeling method. A total of 913 differentially expressed proteins were identified after GC7 treatment (Table S1). Among these proteins, 323 are upregulated and 590 are downregulated (Fig. 3a, b). We next performed Gene Ontology (GO) enrichment analysis of differentially expressed proteins (Figure S5a–c). PANTHER pathway analysis was used to classify the dysregulated proteins. These aberrantly expressed proteins are involved in the integrin signaling pathway, gonadotropin releasing hormone receptor pathway, Ras pathway and others (Fig. 3c). To confirm whether these proteins were specifically regulated by EIF5A1, we performed the iTRAQ assay in HTR-8 cells following transfection with EIF5A1 siRNA and negative control. In all, 945 differentially expressed proteins were identified after EIF5A1 depletion and listed in Supplementary Table 2. By comparing the dysregulated proteins in HTR-8 cells depleted for EIF5A1 and treated with GC7, interestingly, we found that 890 were identical, including 312 upregulation and 578 downregulation (Figure S6; Table S3). Importantly, pathways enriched using PANATHER analysis upon these 890 proteins were same as those upon 913 proteins dysregulated by GC7. These findings suggested that the differentially expressed proteins may be specifically associated with EIF5A1.

Previous studies have reported that the activation of the integrin signaling pathway is important in EVT invasion and embryo implantation^24,25^. We therefore focused on the integrin signaling pathway-associated 10 dysregulated proteins (Fig. 3d). ARAF was 2.04-fold and 3.08-fold downregulated after GC7 treatment and EIF5A1 depletion. Studies have shown that ARAF promotes cell migration, suggesting that it may be a key downstream protein of EIF5A1^26^. We validated the iTRAQ results by western blotting and found that ARAF is significantly decreased in GC7-treated HTR-8 cells (Fig. 3e). In addition, knockdown of EIF5A1 weakens ARAF expression (Fig. 3f). Overexpression of EIF5A1 increases ARAF levels while GC7 attenuates this effect (Fig. 3g). IF staining of ARAF in HTR-8 cells showed results consistent with the western blotting assay (Fig. 3h). Subsequently, we evaluated ARAF levels in trophoblasts in the explant model by IF staining. Consistent with the outcomes in HTR-8 cells, we found that ARAF levels are decreased or increased after EIF5A1 knockdown or overexpression, respectively, and GC7 suppresses ARAF expression both in untreated and EIF5A1 upregulated explants (Fig. 3i). However, mRNA levels of ARAF cannot be regulated by EIF5A1 or GC7 (Figure S7a–c). EIF5A1 has the functions of RNA binding and translation elongation, therefore, we performed RNA-binding protein immunoprecipitation (RIP) assays to study whether EIF5A1 binds to ARAF mRNA. As shown in Fig. 3j–l, the recovery of ARAF mRNA level is decreased from GC7 and siEIF5A1-treated group but increased or not changed in EIF5A1 or EIF5A1_K50A_-transfected HTR-8 cells, compared with their matched controls. It indicates that hypusinated EIF5A1 directly binds to the mRNA of ARAF.

It has been previously reported that, in the absence of EIF5A1, ribosome pauses translocation at polyproline motif resulting in translation suspension and protein downregulation^27^. Recently, researchers revealed that EIF5A1 facilitates translation elongation at not only polyproline, but also at many other motifs (>200 tripeptide motifs in yeast and about 90 tripeptide and pentapeptide motifs in human cells)^28^. We subsequently analyzed the sequences of 590 downregulated proteins following GC7 treatment with UniProt database (https://www.uniprot.org/uniprot/), then found ARAF and other 259 proteins contain those specific motifs (Table S4), suggesting that the translation of their mRNAs may be directly regulated by EIF5A1. Overall, these results verify that ARAF expression is regulated by EIF5A1 in trophoblasts and the regulating mechanism might be associated with translation.

### ARAF expression is decreased in CTBs in RM tissues

**Fig. 4:**
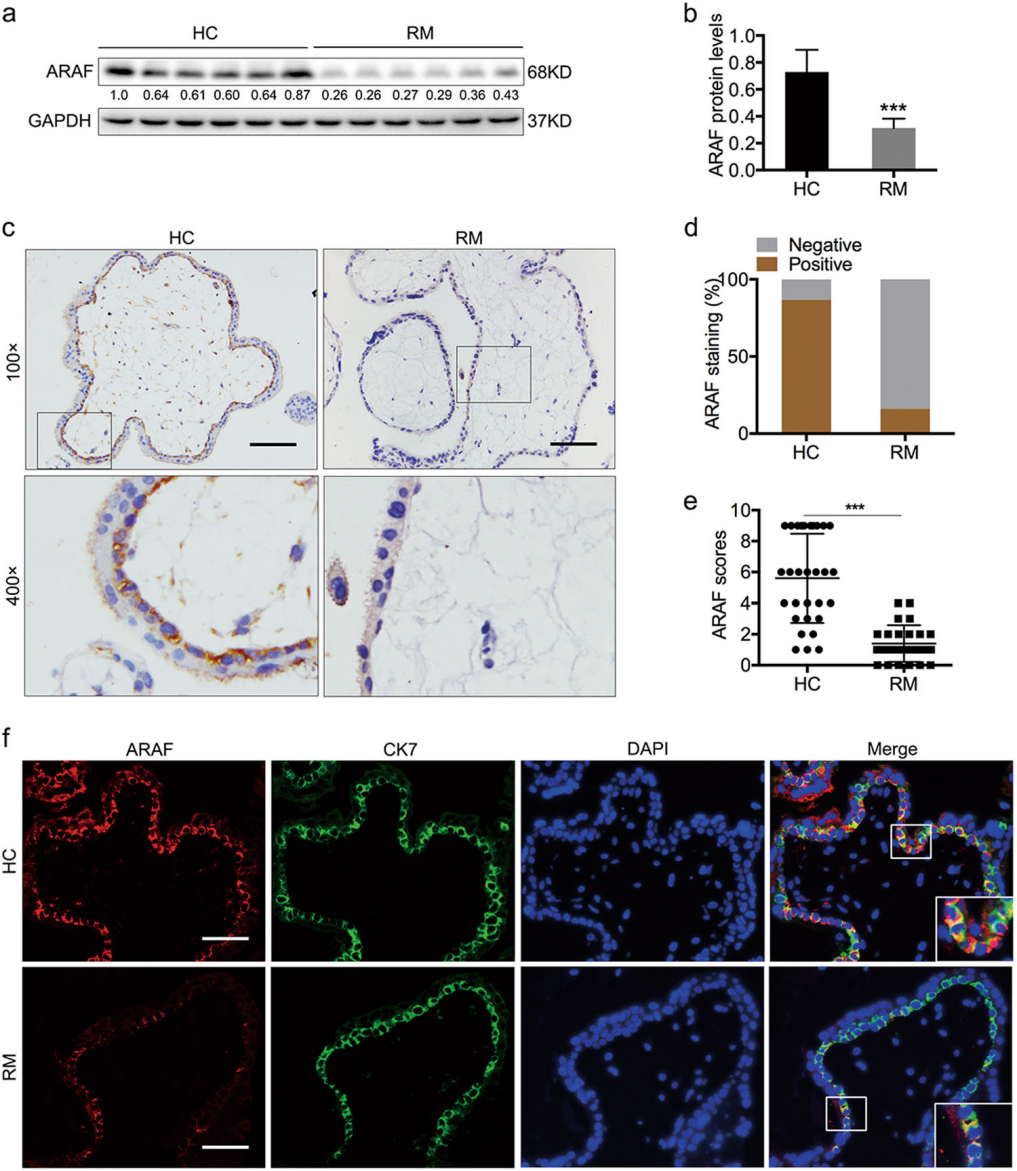
ARAF is downregulated in CTBs of RM tissues. **a** Western blot of ARAF in villous tissues. **b** Relative expression levels of the proteins in (**a**). **c** Representative images of IHC staining of ARAF in 30 HCs and 25 RM samples (scale bar = 100 μm). **d** The percentage of ARAF-positive IHC cases. **e** ARAF IHC scores. **f** Double IF staining of ARAF (red) and CK7 (green) in villous tissues. Magnification × 200, scale bar = 50 μm). ***P < 0.001

Our findings reveal that ARAF is a downstream protein of EIF5A1. We next examined the expression of ARAF in villous tissues. Western blotting assays showed that ARAF is decreased in first-trimester chorionic villous tissues in RM compared with HCs (Fig. 4a, b). To further verify ARAF levels and localization in villous samples, we performed IHC and IF staining of paraffin-embedded tissues (25 RM and 30 HCs). The results show that ARAF staining is mainly localized to the CTB layer and is much stronger in HC than RM tissues (Fig. 4c–f). The Spearman’s rank correlation analysis of EIF5A1 and ARAF according to IHC scores in 25 RM samples suggests that ARAF expression is positively correlated with EIF5A1 expression (Table 1). Double IF staining of EIF5A1 and ARAF in villous tissues shows that higher ARAF fluorescence is always accompanied by stronger EIF5A1 signals (Figure S8a). Together, our results show that ARAF is downregulated in trophoblasts of RM tissues and expression is positively correlated with EIF5A1 expression.

**Table 1:** The correlation between EIF5A1 and ARAF expression levels in 25 RM cases (Spearman’s rank correlation)

	*n*	EIF5A1			
		Negative (*n* = 20)	Positive (*n* = 5)	*r*	*P*-value
ARAF					
Negative	21	19	2	0.519	0.008
Positive	4	1	3		

### EIF5A1 promotes trophoblast migration and invasion through upregulating ARAF expression

**Fig. 5:**
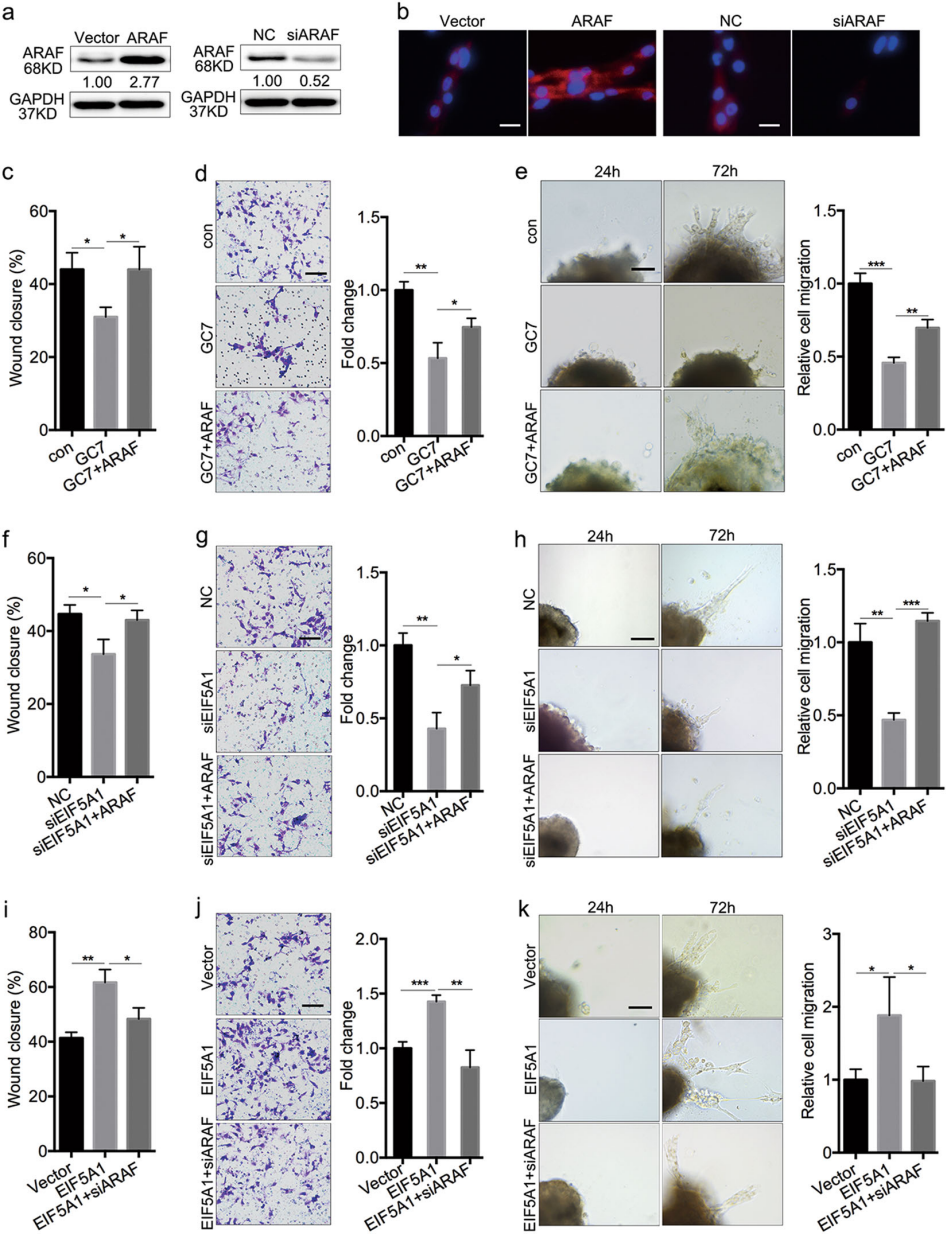
EIF5A1 promotes trophoblast migration and invasion through upregulating ARAF. **a** Western blot showing ARAF levels in HTR-8 cells transfected with siARAF or ARAF. **b** IF staining of ARAF in trophoblasts of villous explants following incubation with siARAF or ARAF. Original magnification × 200, scale bar = 25 μm. **c, d** Effects of ARAF upregulation on migration (**c**) and invasion (**d**) of GC7-treated HTR-8 cells. **e** Effects of ARAF ectopic expression on trophoblast outgrowth in GC7-treated villous explants. **f, g** Changes in migratory (**f**) and invasive (**g**) capabilities in EIF5A1-silenced HTR-8 cells after ARAF overexpression. **h** Effects of ectopic expression of ARAF on trophoblast outgrowth in villous explants incubated with siEIF5A1. **i, j** Changes in migratory (**i**) and invasive (**j**) capabilities in EIF5A1-overexpressed HTR-8 cells after ARAF knockdown. **k** Effects of siARAF incubation on trophoblast outgrowth induced by EIF5A1 overexpression in villous explants. GC7 treatment conditions: 160 μM for 24 h. Wound healing: original magnification × 100, scale bar = 200 μm; Transwell and villous explant culture: original magnification × 200, scale bar = 100 μm. *P < 0.05, **P < 0.01, ***P < 0.001

As EIF5A1 regulates both ARAF expression and the biological behavior of trophoblasts, we examined whether ARAF mediates the regulation of migration and invasion. We transfected HTR-8 cells and villous explants with an ARAF overexpression plasmid or siRNA. Western blotting in HTR-8 cells and IF staining in explants showed that ARAF was upregulated or downregulated after transfection with an overexpression plasmid or siRNA, respectively, compared with negative controls (Fig. 5a, b). Functionally, ARAF overexpression attenuated the inhibitory effects of GC7 on the migration and invasion of HTR-8 cells, as well as trophoblast outgrowth in explants (Fig. 5c–e). In addition, the suppression of wound closure, Matrigel invasion by HTR-8 cells, and the outgrowth of explant trophoblasts caused by EIF5A1 knockdown were reversed by ARAF upregulation (Fig. 5f–h). In contrast, knockdown of ARAF weakens EIF5A1 overexpression-promoted trophoblast migration and invasion (Fig. 5i–k). Therefore, our results confirm that EIF5A1-promoted trophoblast migration and invasion is mediated by ARAF.

### EIF5A1 activates the integrin/ERK signaling pathway via ARAF

**Fig. 6:**
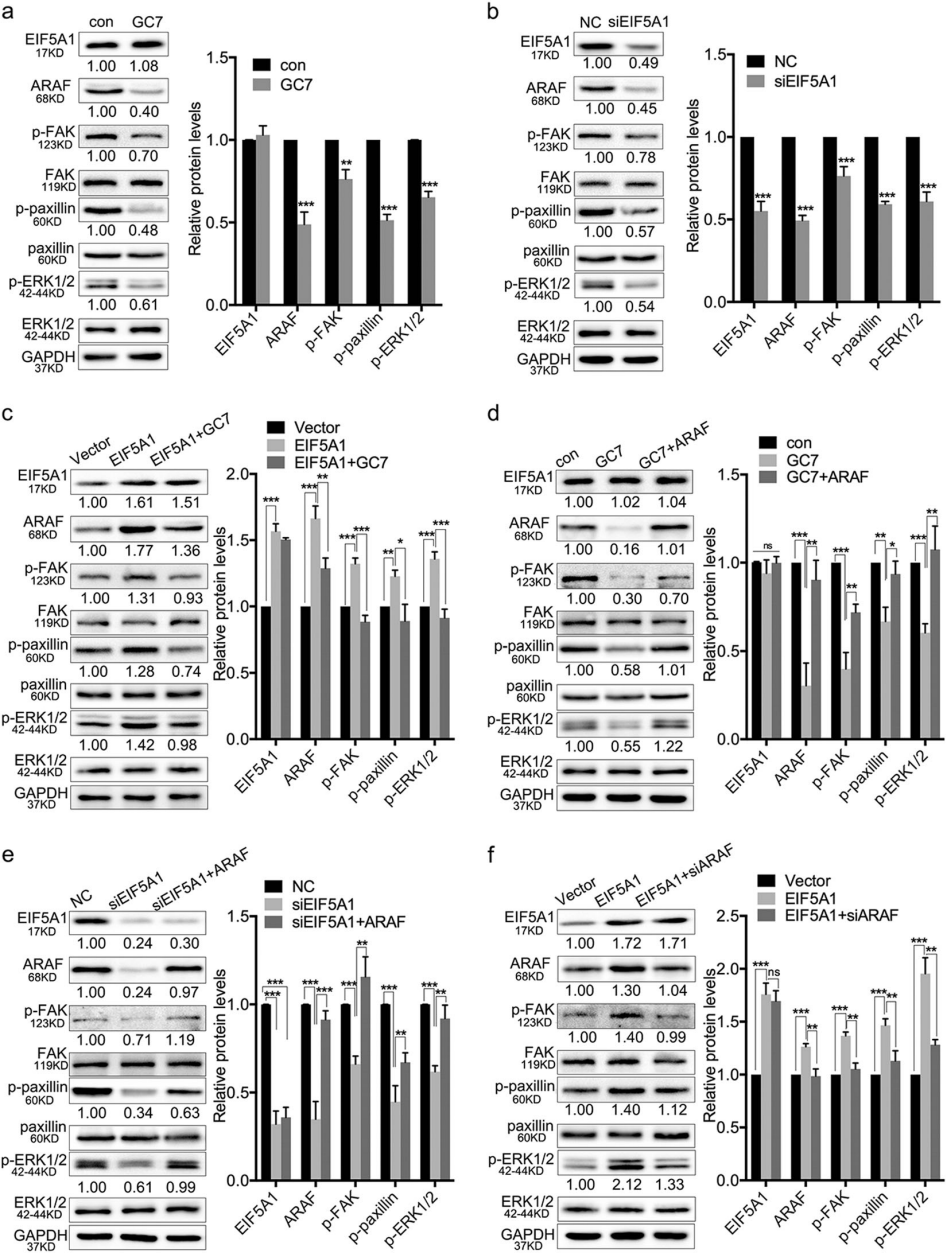
EIF5A1 regulates the integrin/ERK pathway via ARAF. **a, b** Western blot showing the changes in p-FAK, p-paxillin and p-ERK1/2 levels in HTR-8 cells after GC7 (**a**) and siEIF5A1 (**b**) treatment. **c** The effects of EIF5A1 upregulation or GC7 treatment on the expression of p-FAK, p-paxillin and p-ERK1/2 in naive HTR-8 or EIF5A1-overexpressing HTR-8 cells. **d, e** The effects of ARAF overexpression on protein levels in GC7 (**d**) and siEIF5A1 (**e**) treated HTR-8 cells. **f** The roles of ARAF knockdown on protein expression in EIF5A1-overexpressing HTR-8 cells. *P < 0.05, **P < 0.01, ***P < 0.001; ns no significance

The outcome of the iTRAQ labeling assay suggests that EIF5A1 may be a regulator of the integrin signaling pathway. Consequently, we examined the expression of the critical members of the integrin pathway in HTR-8 cells by western blotting to elucidate the underlying mechanisms of RM induced by EIF5A1 downregulation. The results revealed that both GC7 treatment and EIF5A1 knockdown suppress the phosphorylation of FAK, paxillin and ERK1/2 (Fig. 6a, b). Overexpression of EIF5A1 increases p-FAK, p-paxillin and p-ERK1/2 expression, whereas GC7 treatment attenuates these effects (Fig. 6c). Interestingly, after transfection with the EIF5A_K50A_ plasmid, the levels of ARAF, p-FAK, p-paxillin and p-ERK1/2 show no significant difference compared with the vector control group but decrease compared with the EIF5A1 WT group (Figure S9a and S9b). These findings indicate that hypusinated EIF5A1 activates the integrin/ERK pathway.

Western blotting showed that ARAF overexpression attenuates the inhibitory effects of GC7 or siEIF5A1 treatment on the phosphorylation of FAK, paxillin and ERK1/2 (Fig. 6d, e). In contrast, ARAF knockdown reverses EIF5A1-mediated upregulation of p-FAK, p-paxillin and p-ERK1/2 expression (Fig. 6f). Overall, we show that EIF5A1 regulates the integrin/ERK pathway via ARAF and that hypusination of EIF5A1 is necessary for regulation.

## Discussion

The rate of RM is gradually rising along with the increasing age of pregnancy^3,6^. To date, the pathogenesis of RM has not been fully elucidated. In this study, we found that expression levels of EIF5A1 and ARAF are significantly decreased in CTBs of RM samples, suggesting that these two proteins may be involved in the pathogenesis of RM.

Invasion of trophoblasts into the endometrial stroma and inner-third of the myometrium is essential for the development of maternal–fetal circulation and pregnancy success in humans^7,9^. The abnormal proliferative, migratory and invasive capacities of trophoblasts play a vital role in RM^29^. Our study demonstrates that EIF5A1 regulates trophoblasts proliferation, migration and invasion in vitro and ex vivo, which is consistent with the effects of EIF5A1 in other human cells^19–21^. These findings further highlight the role of EIF5A1 in the process of RM. EIF5A1 is unique in containing a hypusinated residue. This modification, found on Lys50, can be inhibited by GC7 (a specific DHS inhibitor), which inhibits most functions of EIF5A1^17^. Herein we found that inhibiting hypusination of EIF5A1 with GC7 suppresses proliferation, migration and invasion both in naive and EIF5A1-overexpressing trophoblasts. Overexpression of the EIF5A1_K50A_, which cannot be hypusinated had no effect on these processes. Therefore, our results confirm that hypusination of EIF5A1 is essential in the regulation of trophoblast biological behaviors including proliferation, migration and invasion.

ARAF is a member of the cytoplasmic serine/threonine kinases Raf family, which are essential factors between Ras and mitogen-activated protein kinase kinase (MEK) in the MAPK-signaling pathway^30^. In comparison with BRAF and CRAF, little is known of the biological functions of ARAF. Some studies suggest that ARAF plays an important role during embryonic development. It was reported that the descendants of ARAF knockout C57Bl/6 mice show intestinal and neurological defects and die between day 7 and day 21 postpartum^31^. Compared with the other two Raf kinases, ARAF has the lowest basal kinase activity (only 20% of CARF). However, ARAF can activate the MAPK pathway in the presence of BRAF and CRAF inhibitors, promoting cell migration^26,32,33^. Herein, we reveal that ARAF is downregulated in trophoblasts in RM tissues and expression is positively correlated with EIF5A1. EIF5A1 promotes trophoblast proliferation, migration and invasion through upregulating ARAF expression. These findings indicate that ARAF is involved in the mechanism of low EIF5A1 levels associated with RM. Above all, we find EIF5A1 could directly bind to the mRNA of ARAF, suggesting a mechanism of translation controlling is involved in the regulation of ARAF expression by EIF5A1. It has been reported that EIF5A1 could facilitate translation elongation through rescuing the ribosome stalling caused by specific motifs^27^, and about 90 tripeptide and pentapeptide motifs are associated with EIF5A1-regulated translation in human cells^28^. By comprehensive analyses, we found that there are three potential motifs (EPP, PPA and VPP) in ARAF sequence, suggesting the translation of it’s mRNA may be directly regulated by EIF5A1. However, which motif is associated with EIF5A1-regulated translation needs more exploration in the future study.

Trophoblast invasion and migration through the uterine wall is mediated by molecular and cellular interactions, controlled by the trophoblasts and the maternal microenvironment^9,34^. ECM proteins such as collagen, laminin, fibronectin and vitronectin are the common components in the microenvironment of maternal–fetal interface. The ECM and associated integrin receptors are involved in trophoblast proliferation, migration and invasion, embryo implantation and the remodeling of the myometrium during pregnancy^9,29,35^. It was reported that the recognition between the endometrium and embryo is mediated by the binding of αvβ3 integrin on the trophoblast cell membrane and its corresponding ligand^36^. The transduction of the integrin signaling pathway is mainly dependent on the activation of FAK and paxillin, then phosphorylation of Ras/Raf and ERK^37–39^. Herein, we found that hypusinated EIF5A1 activates the integrin/ERK pathway through increasing levels of ARAF. These findings reveal that EIF5A1, via ARAF, may be a new regulator of the integrin signaling pathway in trophoblasts. However, the mechanism of EIF5A1-mediated integrin pathway activation remains to be elucidated. One publication indicated that ARAF regulates the phospholipase C gamma 1 (PLCγ1) and phosphatidylinositol 3 kinase (PI3K) signal pathways^40^. Whether this regulation is involved in integrin pathway activation induced by EIF5A1 and ARAF requires further investigation

In conclusion, our study shows that EIF5A1 and ARAF are significantly decreased in CTBs of RM tissues. Hypusinated EIF5A1 promotes trophoblast migration and invasion via ARAF and mediates the activation of the integrin/ERK pathway. EIF5A1 and ARAF may serve as diagnostic markers and therapeutic targets in RM patients.

## Materials and methods

### Patient samples

Between November 2016 and December 2017, 25 patients with RM and 30 healthy pregnant women treated at the Department of Obstetrics and Gynecology in the International Peace Maternity & Child Health Hospital, China Welfare Institute, Shanghai Jiao Tong University School of Medicine were enrolled in this study. Patients with following diagnoses were excluded: (1) uterine malformation or cervical incompetence on pelvic examination and ultrasound, (2) parents or abortus with abnormal karyotype, (3) endocrine or metabolic diseases (e.g., hyperandrogenemia, hyperprolactinemia, diabetes, hyperthyroidism and hypothyroidism), (4) other identified causes of miscarriage. The HCs with previous normal pregnancy terminated their unwanted pregnancies by artificial abortion and had no history of spontaneous abortion, preterm labor or pre-eclampsia. All the RM patients (24–36 years old, mean age 29.64 ± 2.97 years) and HCs (24–36 years old, mean age 29.7 ± 3.88 years) were at 6–10 weeks of pregnancy. All the samples were stored in liquid nitrogen before the extraction of RNA and protein. The Institutional Research Ethics Committee of the International Peace Maternity & Child Health Hospital, China Welfare Institute, Shanghai Jiao Tong University School of Medicine approved this study.

### Cell culture and treatment

The HTR-8/SVneo cell line, which is derived from human invasive EVTs, was a kind gift from Dr. P.K. Lala (University of Western Ontario, London, ON, Canada). The cells were cultured in Dulbecco’s modified Eagle’s medium (DMEM)/F12 with 10% fetal bovine serum (FBS) and maintained at 37 °C in a humidified atmosphere with 5% CO_2_.

GC7 (259545, Merck KGaA, Darmstadt, Germany) was dissolved in 10 mM acetic acid at a stock concentration of 125 mM. One millimolar of aminoguanidine (Sigma-Aldrich, St. Louis, MO, USA) was added to the culture medium to avoid serum amine oxidase inactivation^18^.

The construction of WT EIF5A1 and K50A mutant plasmids and siRNA was previously described^19^. The ARAF overexpression plasmid was ordered from Genechen (Shanghai, China) and siRNA was purchased from RiboBio (Guangzhou, China). Plasmid and siRNA transfections were performed using Lipofectamine 3000 according to the manufacturer’s instructions (Invitrogen, Life Technologies, Carlsbad, CA, USA).

### Western blotting assay

Cells were lysed with radioimmunoprecipitation assay buffer according to standard protocols. The whole-cell protein extract was separated by sodium dodecyl sulfate polyacrylamide gel electrophoresis and transferred to polyvinylidene fluoride membranes. After blocking with 5% non-fat milk, the membranes were cut and incubated with primary antibodies (Table S5) at 4 °C overnight. The membranes were incubated with secondary antibodies at room temperature for 1 h and protein bands detected by enhanced chemiluminescence (Yeasen, Shanghai, China) according to the manufacturer’s instructions. Band densities were calculated with Image J (NIH, Bethesda, MD, USA).

### RNA extraction and real-time PCR

Total RNA was extracted with TRIzol Reagent (Life Technologies, Grand Island, NY, USA) then reverse transcribed using a PrimeScript RT reagent kit (Takara Bio, Kusatsu, Shiga, Japan). Real-time PCR was performed using SYBR Premix Ex Taq (Takara). Relative mRNA levels were calculated using the 2^‒ΔCt^ method normalized to GAPDH. The primers used in this study listed in Supplementary Table S6.

### IF staining

Paraffin-embedded tissues were baked then deparaffinized in dimethylbenzene and rehydrated in a gradient of ethanol. Antigen retrieval was performed in EDTA (pH 8) at 124 °C for 5 min. The samples were incubated in goat serum for 1 h to block nonspecific proteins, then incubated with primary antibody (Table S5) at 4 °C overnight. After a 1-h incubation with the secondary antibody at 37 °C, the nuclei were stained with 4,6-diamidino-2-phenylindole (DAPI). The tissues were observed and images captured by fluorescence microscopy (Lecia DMi8 microscope, Leica Microsystems).

IF staining of cells was described previously^41,42^.

### IHC staining

The IHC staining and scoring was performed as described in our previous work^19^. Briefly, the paraffin-embedded tissues were deparaffinized and rehygrated; antigen retrieval was performed in EDTA then endogenous peroxidase was quenched. The tissues were incubated in goat serum to block nonspecific protein, then incubated with primary antibodies. After incubation with the secondary antibody, diaminobenzidine was added. Then, the tissues were counterstained with hematoxylin and hydrated. Neutral balsam was added, the slides were covered, and images were captured with a microscope. Antibodies used in this study are listed in Table S5.

### Villous explant culture

We conducted villous explant culture as described in our previous study^13^. Briefly, at 8–10 weeks’ healthy gestation, fresh villous tissue was obtained aseptically and dissected into 2–3 mm sections. Five sections per well were cultured in phenol red-free Matrigel-coated 48-well plates with DMEM/F12 plus 10% FBS. After 24 h of culture, the tissues anchored to the surface and began outgrowth. After 48 h, trophoblast outgrowth was observed and pictures were acquired with a light microscope (Lecia).

### MTS, wound-healing and Matrigel transwell assay

We performed the MTS, wound-healing and Matrigel transwell assays as described previously^13,43^.

For MTS assay, 10^4^ cells per well were seeded in 96-well plates. MTS reagent was added and incubated for 0.5–4 h at 37 °C. The optical density (OD) value was measured at 490 nm using a Sunrise Microplate reader (Tecan, Mannedorf, Switzerland).

For wound-healing assay, cells (4 × 10^5^/well) were plated in six-well plates. The monolayers were scraped to create wounds. Images were captured at 0 h and 24 h.

For Matrigel transwell assay, chambers were coated with Matrigel, then inserted into 24-well plates. Culture medium with 20% FBS were added in the bottom chambers. Cells (1 × 10^5^) were plated in the top chamber and incubated with FBS-free culture medium for 24 h. Cells were fixed with 4% paraformaldehyde and stained with crystal violet. Images were captured using a microscope (Leica) and the invaded cells were counted.

### iTRAQ labeling/mass spec assay

HTR-8 cells were cultured in 15 cm dishes then treated with 160 µM GC7 or vehicle for 24 h; EIF5A1 siRNA or negative control for 48 h. Three paired samples were collected and iTRAQ labeling/mass spec assay was performed by TRUMPINC Company (Hangzhou, China). The identified differentially expressed proteins were listed in Tables S1-2.

### RIP assay

RIP assays were performed with the Magna RIP™ Kit (Millipore) according to the instructions. Briefly, the cell lysates from 1 × 10^7^ formaldehyde cross-linked cells were incubated with antibodies against EIF5A1 and IgG at a final dilution of 1:100. Immunoprecipitated RNA was reverse transcribed and subjected to reverse transcriptase-PCR amplification for ARAF.

### Statistical analysis

All data were obtained from three biological and technical replicates and are shown as the mean ± standard deviation (SD). All statistical analyses were conducted with SPSS 21.0 (SPSS Inc., Chicago, IL, USA). *P* *<* 0.05 indicates statistical significance. Student’s *t*-test or one-way analysis of variance were used to compare two groups or multiple groups, respectively. Correlations analyses were performed with Spearman’s rank correlation test.

## Electronic supplementary material

Supplementary Figure Legends

Supplementary Figure S1

Supplementary Figure S2

Supplementary Figure S3

Supplementary Figure S4

Supplementary Figure S5

Supplementary Figure S6

Supplementary Figure S7

Supplementary Figure S8

Supplementary Figure S9

Supplementary Table 1

Supplementary Table 2

Supplementary Table 3

Supplementary Table 4

Supplementary Table 5

Supplementary Table 6
