# Supplementary material for: EIF5A1 promotes trophoblast migration and invasion via ARAF-mediated activation of the integrin/ERK signaling pathway
Source: Cell Death Dis. 2018 Sep 11;9(9):926. doi: 10.1038/s41419-018-0971-5 (PMC6134074; doi:10.1038/s41419-018-0971-5)
Supplement: Supplementary file 1 — Supplementary Figure Legends [file 41419_2018_971_MOESM1_ESM.docx]

**Supplementary Figure Legends**

**Supplementary Figure S1.** **EIF5A1 promotes trophoblast proliferation.** **a** Western blotting of EIF5A1 in HTR-8 cells following transfection with siEIF5A1 or an EIF5A1 expression plasmid. **b** MTS assay demonstrating the effects of EIF5A1 on HTR-8 proliferation. * indicates significance for the vector vs. EIF5A1; # indicates significance for the negative control (NC) vs. siEIF5A1. **P* < 0.05, ***P* < 0.01, ****P* < 0.001.

**Supplementary Figure S2. GC7 inhibits trophoblast proliferation migration and invasion. a** Western blotting assay evaluating EIF5A1 expression after treatment of HTR-8 cells with increasing concentrations of GC7 for 24 h. **b** MTS assay measuring HTR-8 proliferation after GC7 treatment. **c** Matrigel transwell assay showing HTR-8 invasive ability after treatment with GC7 treatment for 24 h. **d** Wound healing assay following GC7 treatment of HTR-8 cells. **e** Trophoblast outgrowth in a villous explant culture model after GC7 treatment.

**Supplementary Figure S3.** **GC7 inhibits EIF5A1-overexpressing trophoblasts migration and invasion *in vitro* and *ex vivo*. a-b** Changes in migratory (**a**) and invasive (**b**) capabilities in EIF5A1-overexpressing HTR-8 cells after incubation with GC7. **c** Effects of GC7 on trophoblast outgrowth induced by EIF5A1 overexpression in villous explants. GC7 treatment was performed at a concentration of 160 μM for 24 h. Wound healing: original magnification × 100, scale bar = 200 μm; Transwell and villous explant culture: original magnification × 200, scale bar = 100 μm.

**Supplementary Figure S4. Transfection with the mutant type EIF5A1_K50A_ plasmid had no effect on HTR-8 cell proliferation, migration or invasion. a** MTS assay measuring HTR-8 cell proliferation after transfection with the vector, EIF5A and EIF5A1_K50A_ plasmids. ***: EIF5A1 vs Vector; ns: EIF5A1_K50A_ vs Vector. **b-c** Changes in migration (**b;** original magnification, × 100, scale bar = 200 μm) and invasion (**c**; original magnification, × 200, scale bar = 100 μm) of HTR-8 cells after ectopic expression of EIF5A1_K50A_. **P* < 0.05, ***P* < 0.01, ****P* < 0.001; ns, no significance.

**Supplementary Figure S5. Functional enrichment of proteins after GC7 treatment based on GO analyses. a** Molecular function. **b** Biological process. **c** Cellular component.

**Supplementary Figure S6. A schematic diagram showing the overlapping differentially expressed proteins originating from two iTRAQ assays.** The left cycle indicates the dysregulated proteins induced by GC7 treatment. The right cycle indicates the differentially expressed proteins caused by EIF5A1 depletion.

**Supplementary Figure S7. ARAF mRNA levels are not regulated by EIF5A1. a-c** The effects of GC7 (**a**), siEIF5A1 (**b**) and EIF5A1 (**c**) treatment on ARAF mRNA expression in HTR-8 cells as evaluated by RT-PCR. ns, no significance.

**Supplementary Figure S8. ARAF expression is positively associated with EIF5A1. a** Representative images of double IF staining of ARAF (red) and EIF5A (green) in villous tissues from RM patients (n=25). Magnification, × 200, scale bar = 50 μm.

**Supplementary Figure S9. Transfection with the EIF5A1_K50A_ plasmid has no effect on the activation of the integrin pathway. a** Western blotting assay showing the protein levels of p-FAK, p-paxillin and p-ERK1/2 in HTR-8 cells after vector, EIF5A and EIF5A1_K50A_ transfection. **b** Relative expression levels of the proteins in (a). ***P* < 0.01, ****P* < 0.001; ns, no significance.
