# Supplementary figures and images for: EIF5A1 promotes trophoblast migration and invasion via ARAF-mediated activation of the integrin/ERK signaling pathway
Source: Cell Death Dis. 2018 Sep 11;9(9):926. doi: 10.1038/s41419-018-0971-5 (PMC6134074; doi:10.1038/s41419-018-0971-5)

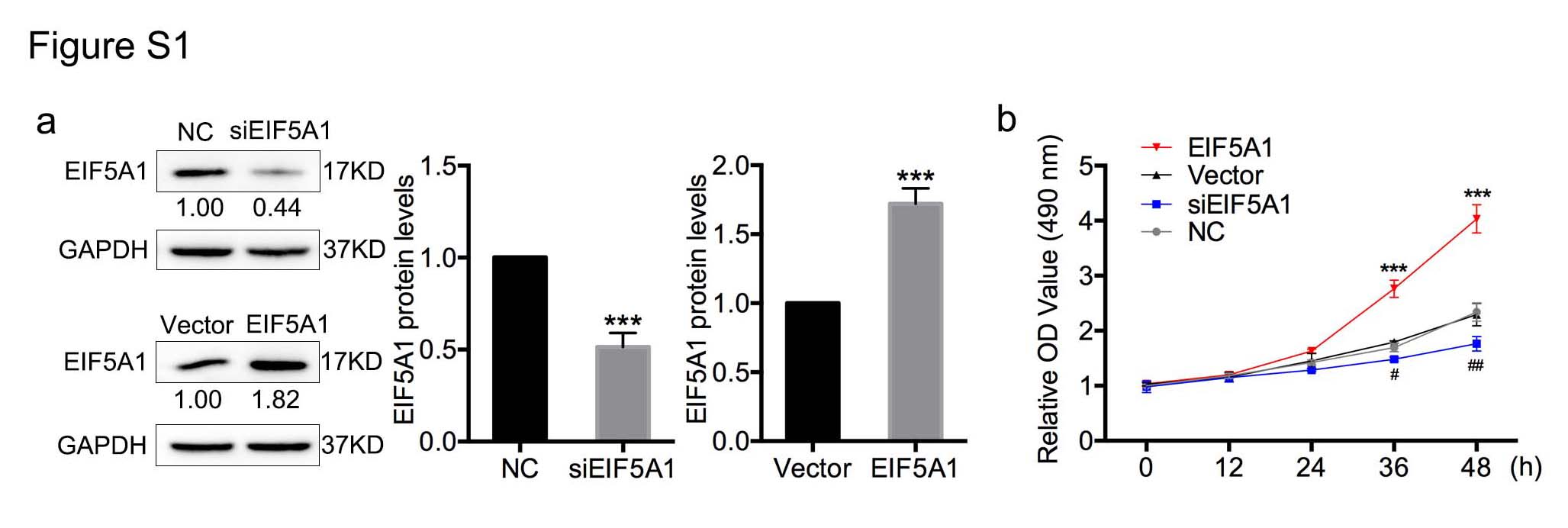

Supplement: Supplementary file 2 — Supplementary Figure S1 [file 41419_2018_971_MOESM2_ESM.jpg]

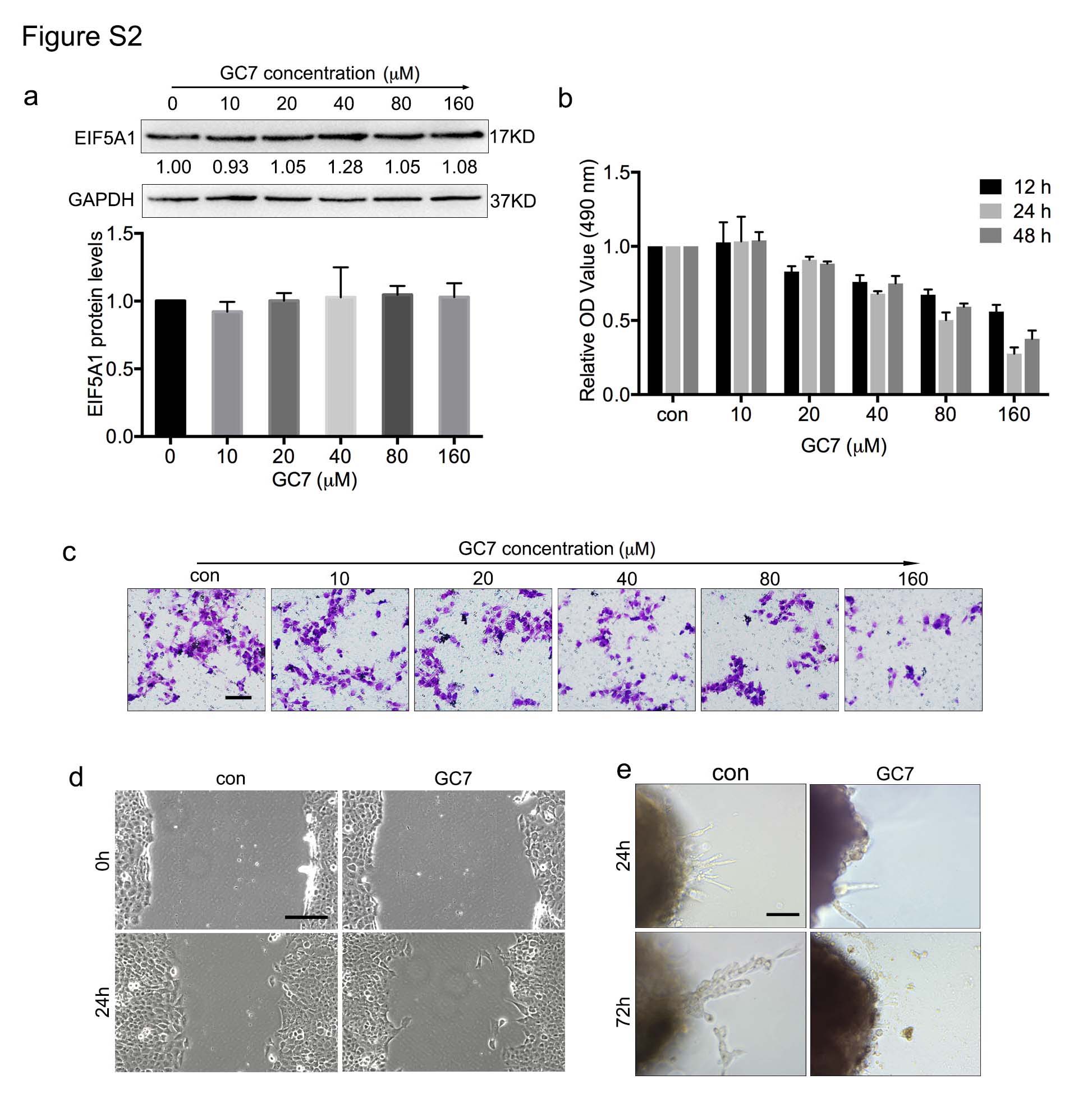

Supplement: Supplementary file 3 — Supplementary Figure S2 [file 41419_2018_971_MOESM3_ESM.jpg]

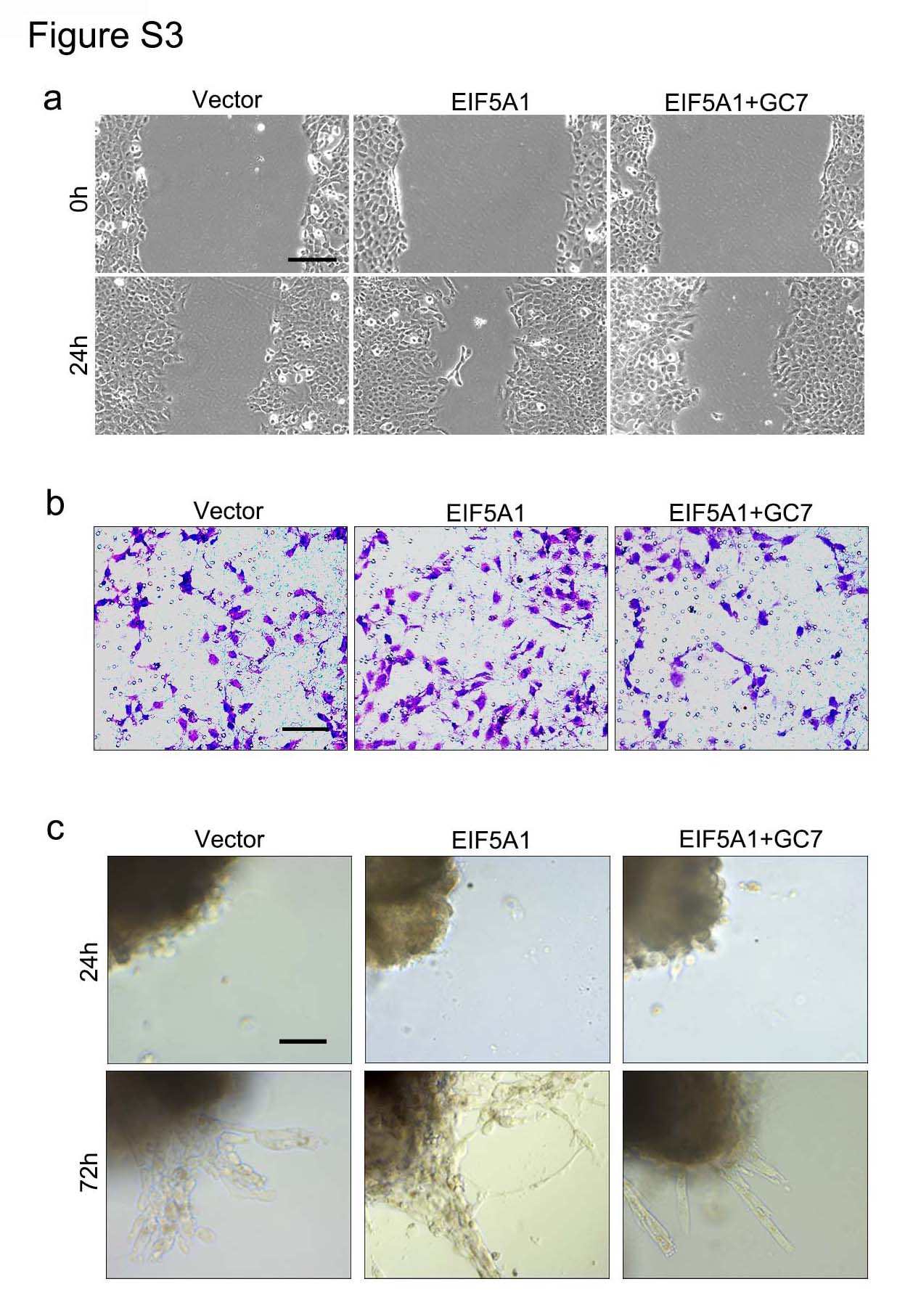

Supplement: Supplementary file 4 — Supplementary Figure S3 [file 41419_2018_971_MOESM4_ESM.jpg]

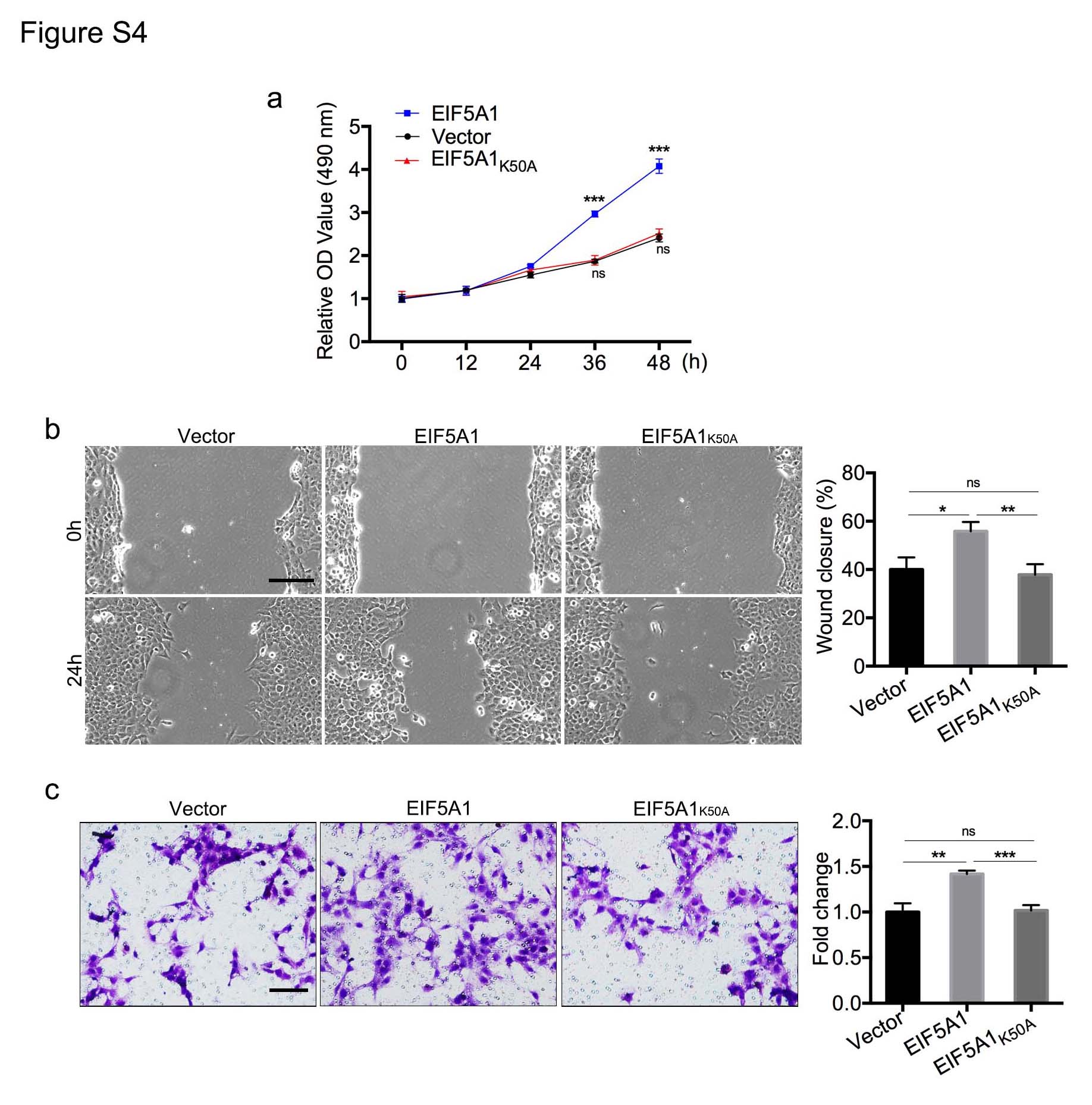

Supplement: Supplementary file 5 — Supplementary Figure S4 [file 41419_2018_971_MOESM5_ESM.jpg]

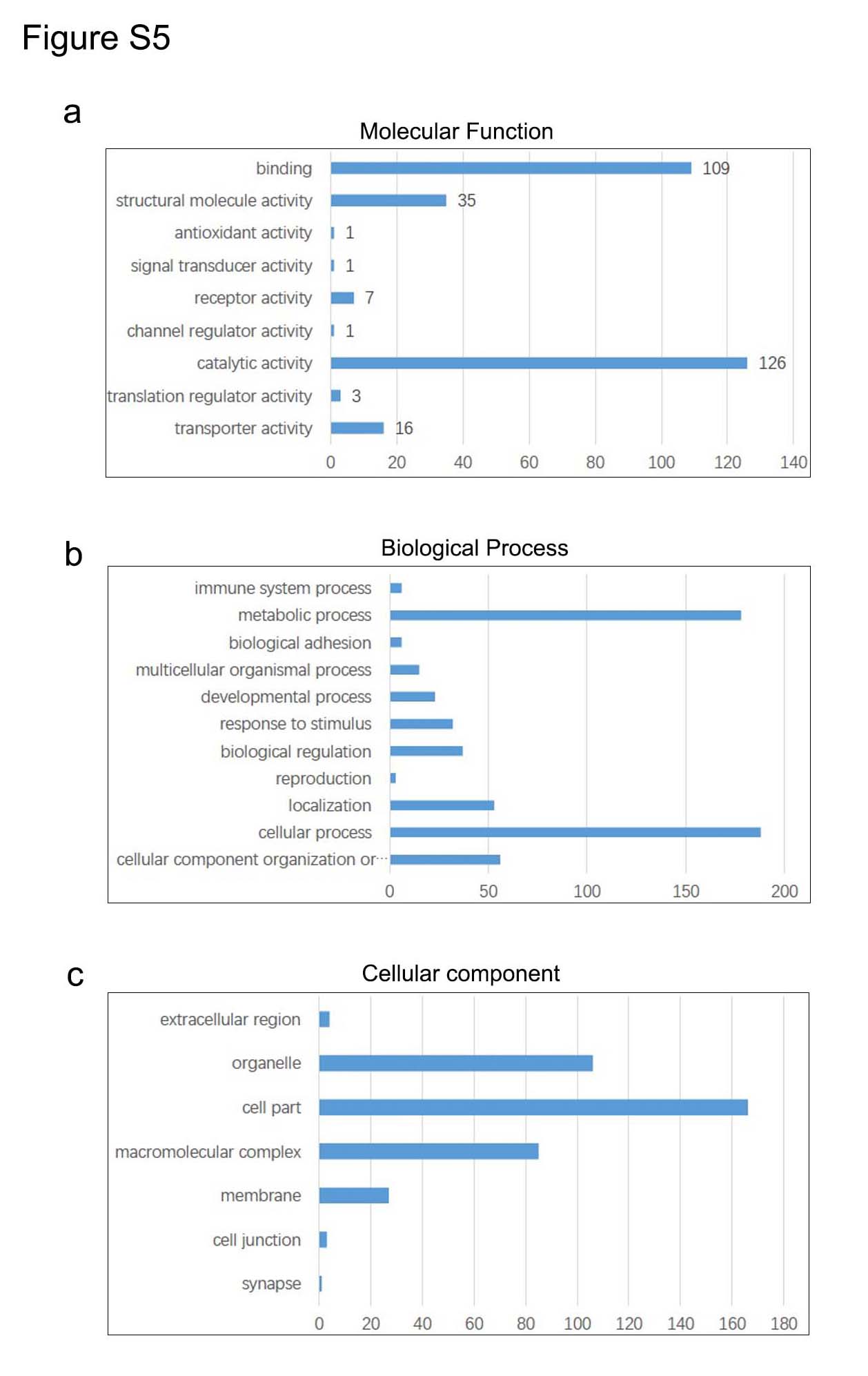

Supplement: Supplementary file 6 — Supplementary Figure S5 [file 41419_2018_971_MOESM6_ESM.jpg]

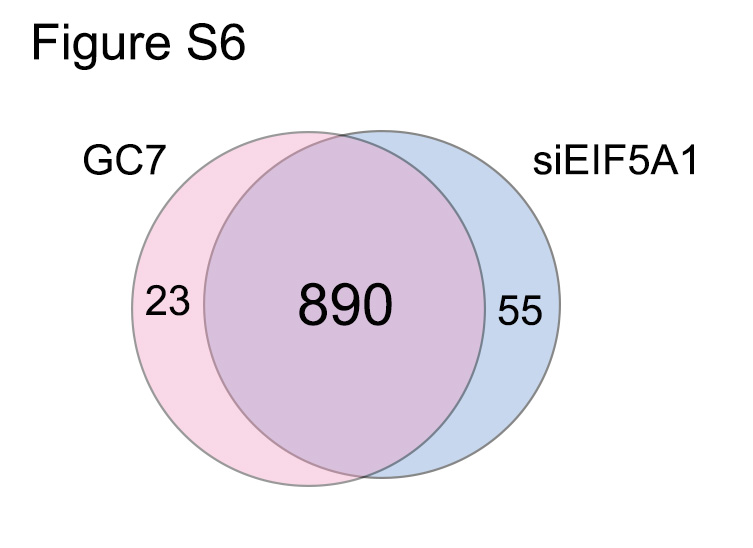

Supplement: Supplementary file 7 — Supplementary Figure S6 [file 41419_2018_971_MOESM7_ESM.jpg]

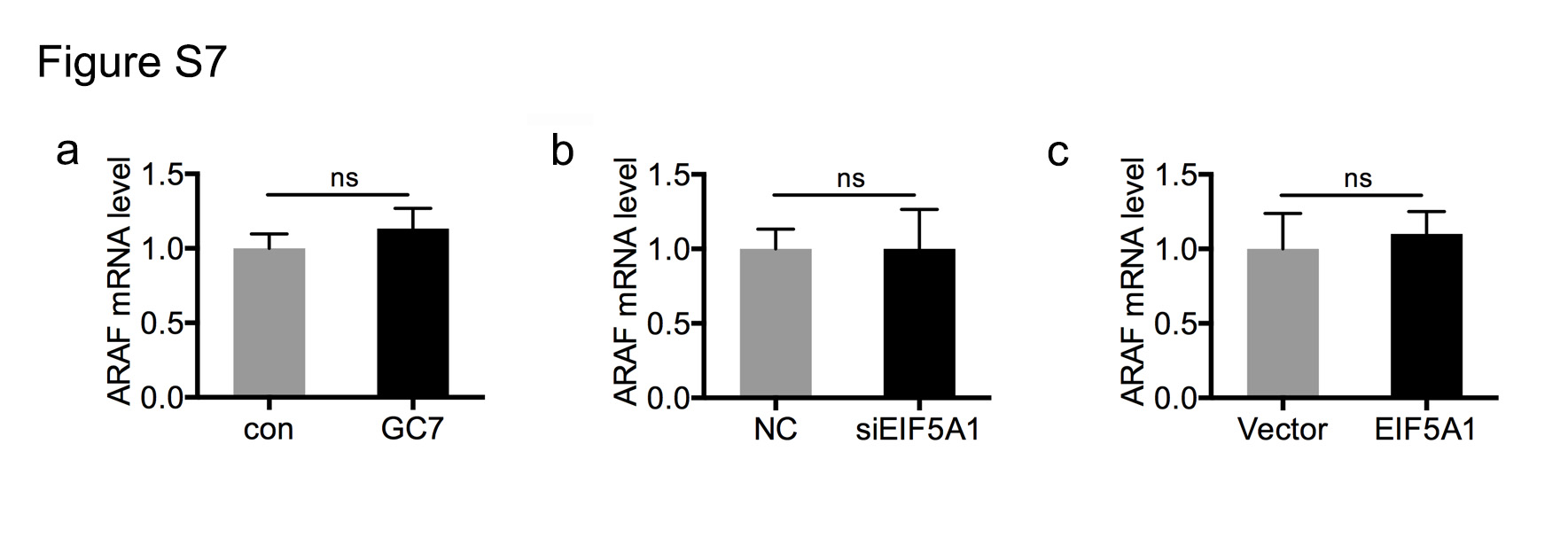

Supplement: Supplementary file 8 — Supplementary Figure S7 [file 41419_2018_971_MOESM8_ESM.jpg]

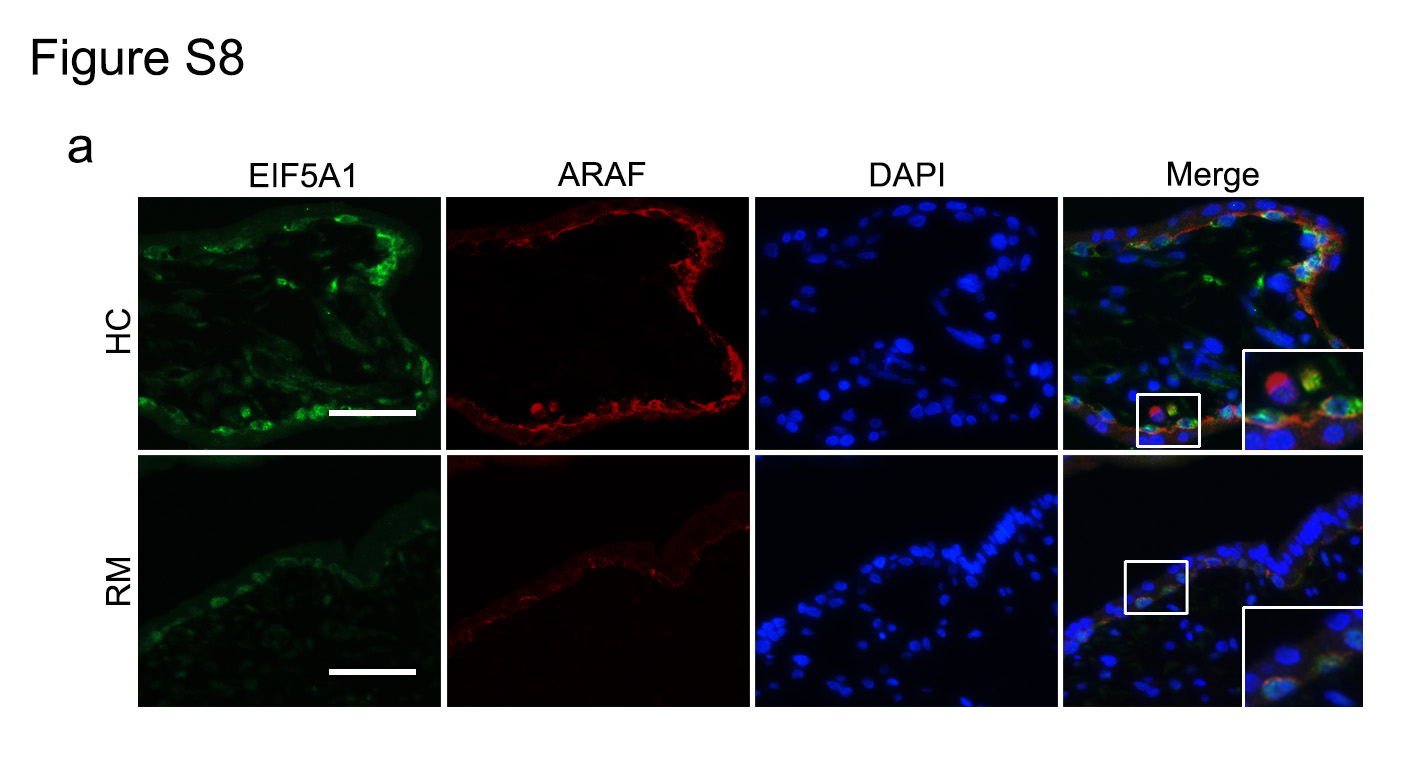

Supplement: Supplementary file 9 — Supplementary Figure S8 [file 41419_2018_971_MOESM9_ESM.jpg]

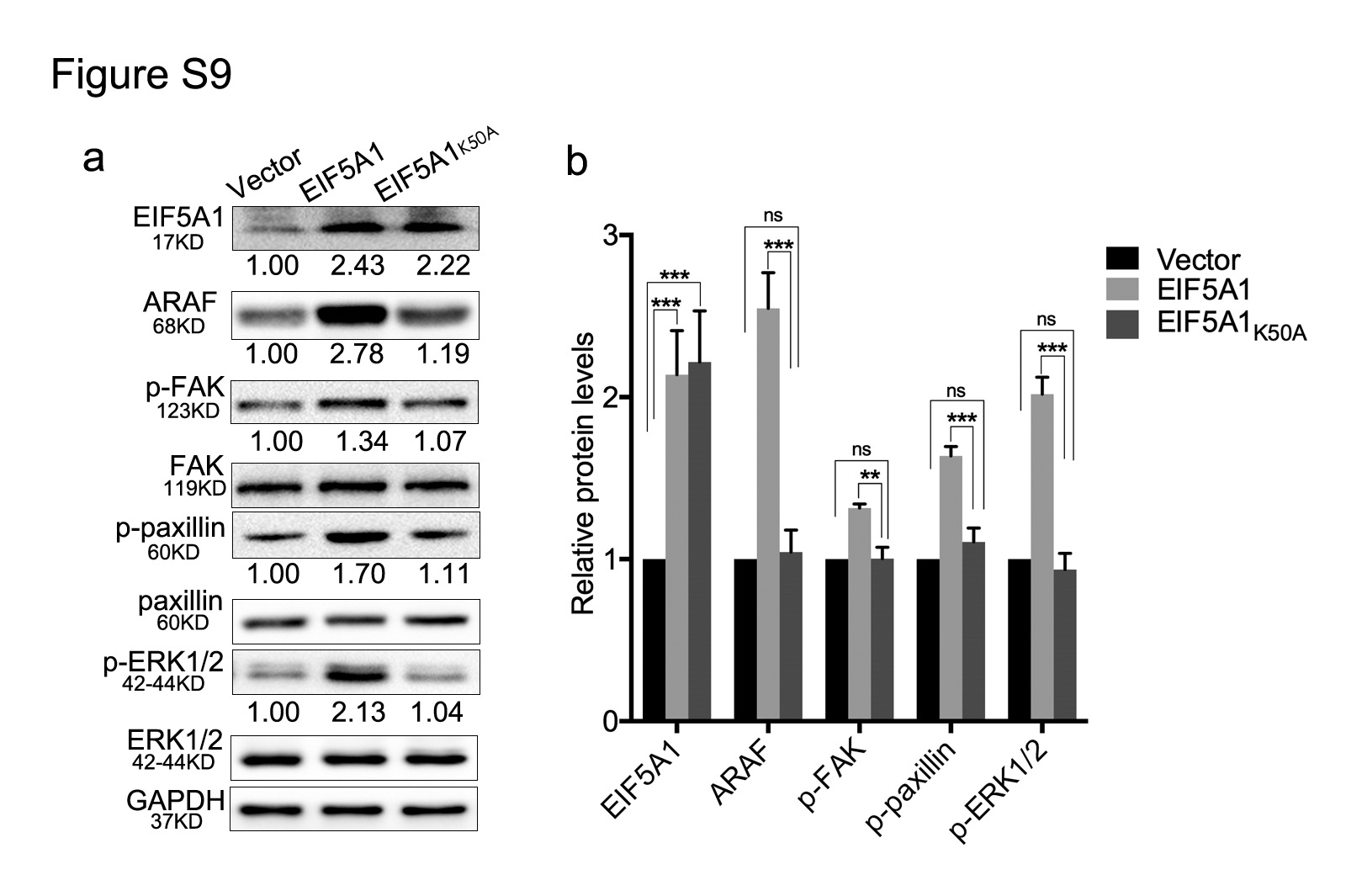

Supplement: Supplementary file 10 — Supplementary Figure S9 [file 41419_2018_971_MOESM10_ESM.jpg]
