# Supplementary material for: EIF5A1 promotes trophoblast migration and invasion via ARAF-mediated activation of the integrin/ERK signaling pathway
Source: Cell Death Dis. 2018 Sep 11;9(9):926. doi: 10.1038/s41419-018-0971-5 (PMC6134074; doi:10.1038/s41419-018-0971-5)
Supplement: Supplementary file 15 — Supplementary Table 5 [file 41419_2018_971_MOESM15_ESM.docx]

**Supplementary Table 5. Antibodies used in this study.**

| Antibody | WB | IHC | IF | Supplier and catalogue number |
| --- | --- | --- | --- | --- |
| EIF5A1 | 1:5000 | 1:500 | 1:500 | Abcam (ab32443) |
| ARAF | 1:1000 | 1:200 | 1:100 | Novus Biologicals (NBP1-33067) |
| GAPDH | 1:10000 | - | - | Abcam (ab181602) |
| CK7 | - | - | 1:200 | Abcam (ab9021) |
| FAK | 1:1000 | - | - | Abcam (ab40794) |
| p-FAK | 1:1000 | - | - | Abcam (ab38512) |
| Paxillin | 1:5000 | - | - | Abcam (ab32084) |
| p-paxillin | 1:1000 | - | - | Abcam (ab4833) |
| ERK1/2 | 1:1000 | - | - | Bioworld Technology (BS1112) |
| p-ERK1/2 | 1:1000 | - | - | Bioworld Technology (BS4621) |

Abbreviations: WB, western blot; IHC, immunohistochemistry; IF, immunofluorescence
