# Supplementary material for: EIF5A1 promotes trophoblast migration and invasion via ARAF-mediated activation of the integrin/ERK signaling pathway
Source: Cell Death Dis. 2018 Sep 11;9(9):926. doi: 10.1038/s41419-018-0971-5 (PMC6134074; doi:10.1038/s41419-018-0971-5)
Supplement: Supplementary file 16 — Supplementary Table 6 [file 41419_2018_971_MOESM16_ESM.docx]

**Supplementary Table 6. The primer sequences used in RT-PCR assays.**

|  | Forward | Reverse |
| --- | --- | --- |
| EIF5A1 | GGCAGATGACTTGGACTTCGAGAC | GCCGTGCTTGCCAGTCTTCG |
| ARAF | CCTGGCGTTCTGTGACTTCTGC | GGCTGTTGGCGGTTGGTACTC |
| GAPDH | TCAAGGCTGAGAACGGGAAG | TGGACTCCACGACGTACTCA |
